# Antimonium-tartaricum

**Published:** 1896-01

**Authors:** Clinton Enos

**Affiliations:** Denver, Col.


### ANTIMONIUM TARTARICUM.

Clinton Enos, M. D., Denver, Col.

This preparation is a compound salt, the Tartrate of Antimony and Potash. The combination seems to be a weak one, or at any rate the action of this remedy is somewhat similar to that of the crude Antimony. Here everything seems to centre about the stomach also. Indeed this feature is so strong that the homœopath hesitates to give Ant.-t. when nausea is absent. Another marked feature is a paretic condition of the chest; this allows exudation to accumulate in the bronchi, because there is an inability to cough up anything on account of this partial paralysis. This chest trouble is present, even when the patient is sick with other complaints. So, in studying this remedy, let us keep these two features in mind. The patient is also generally very irritable, worse at night, in a warm room, and from touch; he is also worse from milk and sour things, and has a white-coated tongue. The general nature of the above statements present to us a true picture of an Ant-t. patient, and his nature should characterize the patient before this remedy is given. We shall now take up each organ of the body to see how it is affected, at the same time keeping in mind the generals that should characterize the patient suffering from any particular symptom of this drug.

In the mental sphere this remedy is as irritable as any remedy known. Children are snappish and cry and whine when touched or looked at.

There is a nondescript vertigo. The headache seems to be of pressive, throbbing character. It is worse in the evening; lying down; getting warm in bed; after eating; sitting bent; and in rest: relieved by sitting upright; in the cold; lying with head high; moving about; and washing the head. This headache is associated with the chest or stomach troubles peculiar to this remedy. We could hardly expect a headache like the above, with the chest and stomach sound, to be cured by

Ant-t., because the nature of the remedy would not probably be present.

The eyes are dim and lose their lustre in a severe case where this remedy is homœopathic. There is that awful appealing look for help coming from the eyes that is seen in cases where life is ebbing away from paralysis of the lungs. This sight, once seen, will never be forgotten. Again, there is a nondescript catarrhal trouble. The books say that it has cured gonorrhœal ophthalmia and old sore eyes of rheumatic and gouty subjects—the other symptoms, of course, agreeing.

The nose, in severe cases, becomes cold and pointed; the alæ nasi flap as in Lyc.; the inside of nostrils is black and sooty.

Despairing anxiety is depicted upon the face. At first it may be flushed, but it soon assumes a bloated, livid look, or a drawn, ashy whiteness, as is seen in Carbonic-acid gas poisoning. In fact, this is what is the matter with the patient, for the lungs are so weak and so filled with mucus, as we shall see hereafter, that the blood is not properly oxygenated. At other times the face is cold and pale; lips purple; skin bluish around nose and eyes.

The tongue in this remedy is similar to that of Ant-cr. It has a thick white coat. Occasionally the coating is not so thick and the reddened papillæ show up through it. Rarely the tongue is brown. The taste is bitter; sometimes flat.

There is a loss of appetite, with a disgust for food. Thirstlessness is the rule; when thirst is present the patient desires cold water.

Eating sour things or drinking sour wine makes the patient worse; also, milk disagrees.

In diseases of the intestinal tract there is great nausea with intense anxiety. There is retching and vomiting of green and watery matter, sometimes frothy or mucous; drowsiness after vomiting. In children there is vomiting of food as soon as taken. Sometimes in summer complaint there is great thirst for cold water. There may be vomiting and purging at the same time, with every symptom of collapse; coldness of the surface; the hands and feet are like ice, and the stools are profuse and watery. When complaints are not very severe there is belching of gas, having a foul taste, or tasting of rotten eggs, which gives relief to the patient. With this trouble you are apt to find that there is rattling in the throat of mucus with an inability to get it up. This does not come on, however, unless the case gets very severe.

There is a diarrhœa of nondescript stool with sharp cutting in abdomen, just before stool. Just this much would not be an indication for any remedy, but if Ant-t. is to be given there must be present, continuous, anxious nausea, straining to vomit with perspiration on forehead and face; after vomiting there is great drowsiness and languor; eructations smelling of rotten eggs; white tongue; thirstlessness or thirst for cold drinks; aversion to milk and some acute cases crave acids; irritability. At first some other remedy may be given, but if improvement does not take place, and the child begins to sink and get that rattling in its throat, and assumes that deathly pallor and distressing look, you can readily see that the wrong remedy has been given. Ant-t. will save this child, even after all hope for its recovery has been abandoned.

In the male sexual system this remedy has been used to cure the pain and swelling in testicles, caused by suppression of gonorrhœa. It has also cured figwarts behind the glans penis. So you see that there is something of a sycotic nature in Tartar-emetic. There are pustules on the genitals as a result of this remedy. These may prove similar enough to chancres for this remedy to be used against syphilis. Of course the generalities of the patient must correspond to the generalities of Ant-t. or the remedy will not be homœopathic.

Irregularities of the menstrual function are so vague that the gastric derangement and chest symptoms afford better indications for Ant-t. in this kind of trouble than do the menstrual symptoms themselves.

During pregnancy there is gastric derangement; vomiting of mucus; belching; disgust for food; salivation; nausea, with faintness; amblyopia after colic or strong emotions; oppression of chest; loose, rattling cough; no sputa.

The symptoms of Carbonic-acid gas poisoning have led to the use of this remedy, in asphyxia, of the new-born child.

Now let us take up the respiratory tract and study the symptoms as they appear in this part of the body. There is great rattling in trachea and chest. The breathing is abdominal on account of the paresis of the lungs. The abdomen heaves and sinks like that seen in dying persons sometimes. In pneumonia, whooping-cough, and bronchitis there is difficult breathing with loud rattling in chest and trachea, and in many cases can be heard across the room. You think if he would cough a little he would get the mucus up; he does cough but nothing comes up, on account of the paretic condition of the respiratory muscles. Hence he uses the abdominal muscles in breathing, to make up for the loss sustained by the weakness of the chest muscles. The cough is so feeble that when you hear it you feel inclined to help him by coughing vigorously yourself. His face is drawn and cold; nostrils dilated and sooty on the inside. With this there is nausea; he is more likely to get up a little mucus when he vomits than when he coughs. Now this state is hardly one you would expect in the first days of bronchitis, etc., but one that would come about after several days of sickness, when there is threatened paresis of lungs, with Carbonic-acid gas poisoning. The cough is worse at 11 P. M., toward morning; after eating; from getting angry, and getting warm in bed: relieved by sitting upright. With all this there must be present the generalities of this remedy.

Sometimes we see children taken down suddenly with bronchitis, and there is that rattling with inability to get up mucus; in some cases they spit it up, but it forms faster than it can be coughed up; there is also the nausea, etc., as described above. This is not an Ant-t. case, because the rattling comes during the period of irritation, and not as a result of paresis of the chest. Ipec. fits this condition. These two cases may seem somewhat similar superficially, but their natures are entirely different.

Whooping-cough: child cannot bear to be touched or looked at ; white tongue ; great rattling in chest ; coughs when he eats or gets angry—the latter he does very often.

Asthma, with difficulty to exhale ; hydrothorax ; hypostatic congestion of lungs ; together with the generalities of the remedy.

Ant-t. exerts a depressing influence upon the heart and circulation. During lung and stomach troubles the heart is disturbed and often becomes inflamed, especially pericarditis. This remedy has been used for cyanosis from organic heart disease, but, as far as I know, has not been used, especially for old organic trouble of the heart.

This remedy cures lumbago when the slightest effort to move causes nausea, retching, and cold, clammy sweat ; white tongue, etc., also present.

There is an irresistible inclination to sleep with nearly all complaints.

Chill, fever and sweat with no thirst in any stage, but predominant gastric symptoms, are characteristic of this remedy.

Acute articular rheumatism also comes within the range of this remedy. The joints are somewhat swollen, reddened, hot, and very painful at every attempt to move them. The general nature of Ant-t. must be present or there is no use to give it, for some other remedy will be indicated.

On the skin there are pustules as large as a pea. They become umbilicated, scab over and leave a scar. So when you have a case of small-pox, and have the chest and stomach symptoms of this remedy present, or anything else that will show forth the general nature of this remedy, you may give it and your prescription will be homœopathic.

This article has been written to bring out the general nature of Ant-t. When we once get the general nature of a remedy understood, we can soon fill out particulars and thereby become proficient in our knowledge of it, and so become masters of our materia medica.

